# “One-stop shop”: safety and efficacy of combining atrial septal defect occlusion and left atrial appendage closure for patients with atrial septal defect and atrial fibrillation

**DOI:** 10.1186/s12872-020-01708-6

**Published:** 2020-10-12

**Authors:** Zhi-hui Zhang, Qing Yao, Hai-yun Huang, Ping Zhu, Xiang Xu, Zhi-Yuan Song, Hua-kang Li

**Affiliations:** 1 Department of Cardiology, Southwest Hospital, Army Medical University, Chongqing, China; 2 Department of ultrasound, Southwest Hospital, Army Medical University, Chongqing, China

**Keywords:** Atrial fibrillation, Atrial septal defect, Patent foramen ovale, Left atrial appendage closure

## Abstract

**Background:**

One-stop occlusion, which is defined as the combination of atrial septal defect [ASD] or patent foramen ovale [PFO] occlusion and left atrial appendage [LAA] closure, in patients with ASD/PFO and atrial fibrillation (AF) has not yet been investigated systematically. This study aimed to evaluate the safety and efficacy of one-stop occlusion in the treatment of adult patients with ASD/PFO and AF.

**Methods:**

Inpatients with AF and ASD/PFO were recruited between August 2014 and April 2019. Preoperatively, transthoracic echocardiography (TTE) and transesophageal echocardiography (TEE) were conducted to identify the ASD/PFO size and margin, presence of thrombus in the LAA, and LAA orifice width and depth at 0°, 45°, 90°, and 135°. After confirmation of the indications of LAA closure (LAAC) and ASD/PFO occlusion, the procedures were performed simultaneously under general anesthesia. Oral anticoagulants were administered for 45–60 days, followed with regular evaluation of TTE and TEE.

**Results:**

Forty-nine patients (age, 65.6 ± 9.6 years) were recruited in this study, including 24 patients with ASD and 25 patients with PFO. They were treated with LAAC and ASD/PFO occlusion successfully. The mean ASD size and mean diameter of the ASD occluders were 14.2 ± 7.7 and 25.4 ± 8.5 mm, respectively. The mean PFO size was 3.5 ± 0.4 mm. The mean maximal LAA orifice width and depth were 20.5 ± 3.4 and 28.3 ± 3.6 mm, respectively. All patients were implanted with a Watchman device (diameter, 27.1 ± 2.9 mm). Postoperatively, all patients took anticoagulants orally for 45–60 days, and their mean postoperative follow-up duration was 29.0 ± 12.1 months. Postoperative TEE showed that all had normal positioning of the LAA and ASD/PFO occluders. At 45–60 days after operation, TEE showed that the LAA and ASD/PFO occluder were in the normal position; however, two patients who took warfarin and novel oral anticoagulants, respectively, have developed occluder thrombosis. After adjusted anticoagulant therapy, TEE showed that the thrombus disappeared at 6 months after operation.

**Conclusion:**

One-stop occlusion is safe and effective for the treatment of adult patients with ASD/PFO and AF. It is also feasible to administer warfarin or novel oral anticoagulants after operation.

## Background

Atrial septal defect (ASD) is clinically common, accounting for 20–30% of congenital heart disease cases [1–3]. Conversely, patent foramen ovale (PFO) refers to the condition in which the foramen ovale does not close after 3 years of age. Clinical research has confirmed that PFO is closely related to migraine and abnormal stroke [4, 5]. With the continuous development of interventional therapeutic devices for congenital heart diseases, interventional occlusion of secundum ASD/PFO has been gradually improving. Atrial fibrillation (AF) is a clinically common tachyarrhythmia that can lead to cardiac insufficiency and thromboembolism [6]. Given that > 90% of thrombi in patients with AF and non-valvular heart disease arise from the left atrial appendage (LAA), transcatheter LAA closure (LAAC) has become an important approach for preventing the occurrence of thrombus in such patients. Its clinical benefits and safety have been reported in many randomized controlled trials and cohort studies [7–9].

Previous studies have shown that the occurrence rate of AF in patients with ASD is significantly higher than that in normal individuals and increases proportionally with age [10, 11]. The literature indicates that the occurrence rates of AF in patients with ASD aged > 40 and > 60 years are approximately 21 and 52%, respectively [12–14]. ASD shunts in the atrium allow blood flow from the left atrium to the right atrium, causing the blood volumes of the right atrium, right ventricle, and pulmonary artery to increase, thus aggravating the right ventricular volume overload. The pathological basis of AF in patients with ASD is enlargement of the right atrium and right ventricle. Radiofrequency catheter ablation (RFCA) has a lower success rate and higher recurrence rate for these patients. At present, the procedure of ASD/PFO occlusion and LAAC are gradually mature. However, for patients with ASD/PFO and AF, one-stop occlusion (combination of ASD/PFO occlusion and LAAC) has not yet been investigated systematically, and its feasibility is unknown. In this study, 49 patients with ASD/PFO and AF underwent one-stop occlusion, and their clinical data were analyzed to assess the feasibility of the treatment.

## Methods

### Patient selection

Inpatients with AF and ASD/PFO at the Southwest Hospital of the Army Medical University were included between August 2014 and April 2019, based on the following inclusion and exclusion criteria. Inclusion criteria: a. For patients with PFO, they have ischemic stroke, or transient ischemic attack (TIA), or a peripheral thromboembolic event or a large right-to-left shunt. For patients with ASD, TTE show clear indications for ASD occlusion. b. TEE show clear indications for LAAC. c. Non-valvular AF. d. CHA2DS2VASc score ≥ 2, and HAS-BLED score ≥ 3 or having a contraindication to anticoagulant therapy or unwilling to receive long-term warfarin therapy. e. Patients were willing to accept the one-stop occlusion. Exclusion criteria: a. Valvular heart disease. b. Left atrial or LAA thrombosis. c. ASD requiring surgical repair, congenital heart diseases requiring other thoracotomy treatment. d. Severe heart failure (New York Heart Association class IV). e. Severe renal or hepatic insufficiency. f. Acute stroke occured within 1 month.g. Patients were unwilling to accept the one-stop occlusion. This was a retrospective study. Ethical approval of this study was obtained from ethics committee of Southwest Hospital (the approval number was KY2020171 and the date was March 92,020). All the participated patients provided written informed consent.

### Devices

The LAAC devices adopted in this study were the Watchman LAA Closure Device and Access Sheath produced by American Boston Scientific. ASD occluders and delivery systems were produced by Shanghai Shape Memory Alloy Materials Co., Ltd. The PFO occluders and its transmission system were manufactured by Beijing Huayi Shengjie Technology Co., Ltd.

### Preoperative preparation

**Fig. 1:**
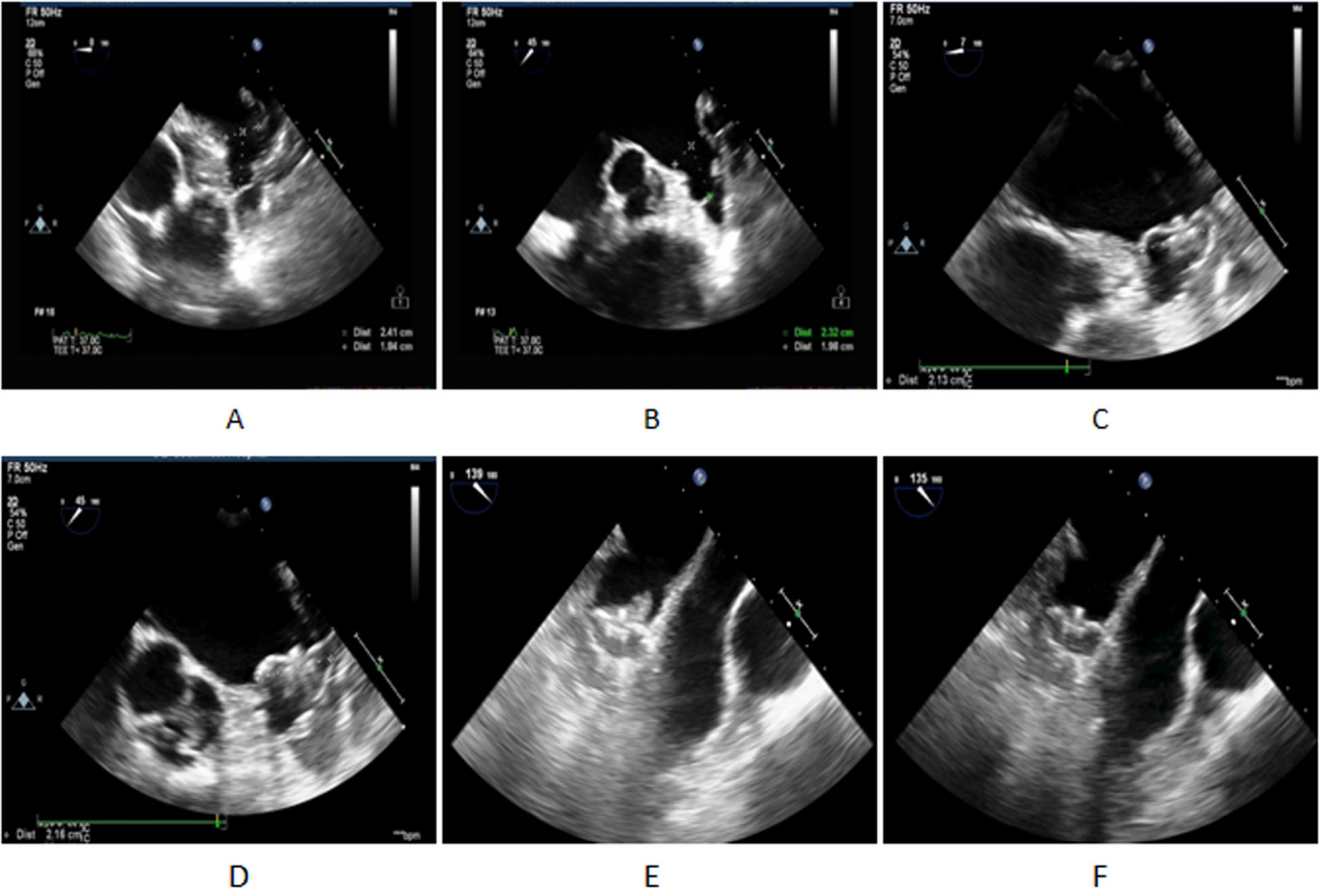
TEE imaging of successful LAAC and a double ASD patient with persistent AF (Measurements of the LAA orifice width and depth from 0°(**a**) and 45°(**b**); after LAAC,TEE shows the LAA occluder from 0°(**c**) and 45°(**d**); Thrombosis in LAA occluder(**e**); Thrombosis dispeared in LAA occluder(F)

The patients were examined preoperatively, including routine examination of the blood, coagulation function, hepatorenal function, thyroid function, ECG and pulmonary function test, TTE, and TEE. The ASD/PFO size and margin were measured. The LAA morphology was observed, and the presence of thrombus was determined. The LAA orifice width and depth were measured at four angles, 0°, 45°, 90°, and 135°, to confirm whether the patients had LAAC (Fig. 1a and b).

### LAAC

**Fig. 2:**
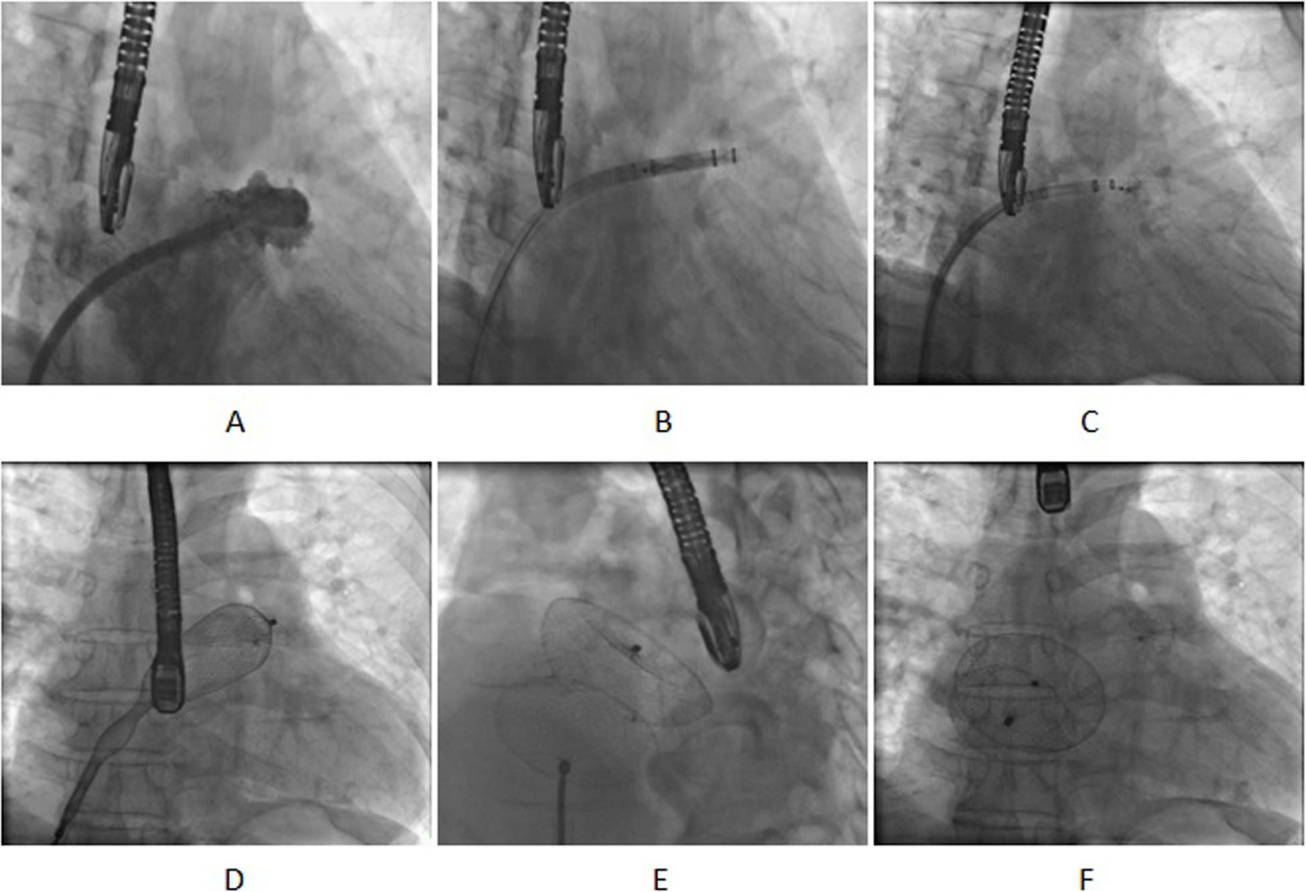
X-ray imaging of the process of LAAC and ASD occlusion (LAA angiography(**a**), LAA occluder were sent into the LAA(**b**), A pull test was conducted to judge the stability of the LAA occluder(**c**), ASD/PFO plug devices were sent into left atrium(**d**), A pull test was conducted to judge the stability of the ASD occluder(**e**), the LAA and ASD occluder were released(**f**)

Under general anesthesia and TEE monitoring, the patients underwent LAAC [15]. The right femoral vein was punctured. Right heart catheterization was performed to measure the pressure in the pulmonary artery, right ventricle, and right atrium. A catheter was inserted through the ASD and into the left atrium to measure the pressure. To perform LAA angiography (Fig. 2a), a special sheathing canal of the LAAC was placed, through which a pigtail angiographic catheter was directed to the LAA with the following positions: Right Anterior Oblique (RAO) 30° + Cranial (CRA) 20° and RAO 30° + Caudal (CAU) 20° (Fig. 2a). LAA orifice width and depth measurements, in combination with TEE measurement, were used to select suitable LAA occluders, with the diameter of the Watchman devices determined to be ≥4-6 mm, larger than the LAA. The LAA occluders were sent into the LAA along the sheathing canal for LAA angiography (Fig. 2b). TEE was used to observe the position of the occluders and their effects. A pull test was conducted to judge the stability of the LAA occlude (Fig. 2c). After confirming suitable positioning of the LAA occluders and good plugging effect, the occluders were released (Fig. 1c and d), and the pressure in the left atrium was measured repeatedly.

### ASD/PFO occlusion

ASD/PFO occlusion was performed in accordance with the routine method used during catheterization [16]. A special sheathing canal for the ASD/PFO occlusion was placed in the left atrium. ASD/PFO occluders were sent along the sheathing canal to plug the ASD/PFO (Fig. 2d). Under X-ray and TEE guidance, a pull test was performed to observe the stability of the occluders and their effects (Fig. 2e). After confirming that the occluders were suitably positioned, they were released (Fig. 2f). The sheathing canal was withdrawn.

### Postoperative treatment

After the operation, patients stayed in the cardiac care unit for 24-h continuous monitoring of ECG, blood pressure, and oxyhemoglobin saturation. Low-molecular-weight heparin was subcutaneously injected after operation. Oral warfarin or Novel Oral Anticoagulants(NOAC) was administered at 45–60 days after operation. If the TEE showed complete closure of the LAA, no device-related thrombus, the patients were then switched to both aspirin and clopidogrel until 6 months. The INR was maintained at approximately 2.0–3.0. After 6 months, they began taking enteric coated aspirin tablets (100 mg) once per day.

### Follow-up visit

Patients underwent follow-up visits at 3, 30, 45–60, 90, 180 and 360 days after operation. During the follow-up visits, the patients reported their subjective symptoms, and their blood pressure, heart rate, ECG, and TTE were recorded. During the first 45–60 days of follow-up visit, TEE was performed. The position of the plug device was checked for any shifting. The patients were also checked for remnant shunts and relevant thrombogenesis of devices. Their INR was checked, and their warfarin dosage was adjusted accordingly. The patients were followed up again every 6 months.

### Statistical analysis

SPSS 13.0 statistical software (SPSS, Chicago, IL, USA) was used. Continuous data were expressed as mean ± standard deviation. Analysis of variance and Chi-square test were adopted to compare the data between the groups. The counted data were analyzed by using the χ2 test. In all tests, *P* values < 0.05 were considered statistically significant.

## Results

### Patient characteristics

In the ASD group, there were 24 patients with ASD and AF (two patients with double-hole ASD); the average ASD diameter was 14.5 ± 7.4 mm, and the types of AF were persistent AF in 21 patients and paroxysmal AF in 3 patients. Five patients are unwilling to receive long-term warfarin therapy. In the PFO group, there were 25 patients with PFO and AF, including 24 patients with persistent AF and 1 patient with paroxysmal AF. Six patients are unwilling to receive long-term warfarin therapy. Table 1 shows the basic data and clinical characteristics between the two groups.

**Table 1:** Patient characteristics

Patient characteristics	ASD group(*n* = 24)	PFO group(*n* = 25)	P
Sex (Male)	11/24	11/25	0.90
Age (year)	64.8 ± 9.7	66.3 ± 9.7	0.57
BMI (Kg/m^2^)	23.3 ± 4.3	24.9 ± 3.0	0.14
AF type			0.35
Persistent AF	21	24	
Paroxysmal AF	3	1	
AF time	3.9 ± 3.5	3.8 ± 4.9	0.94
Hypertension	9	11	0.64
Diabetes Mellitus	6	7	0.81
Stroke/TIA	4	5	0.76
CHA_2_DS_2_VASc score	3.3 ± 0.7	3.6 ± 0.9	0.14
HASBLED score	2.5 ± 0.6	2.6 ± 0.6	0.55
NYHA			0.003
II class	13	23	
III class	11	2	

### Interventional therapeutic outcomes

**Fig. 3:**
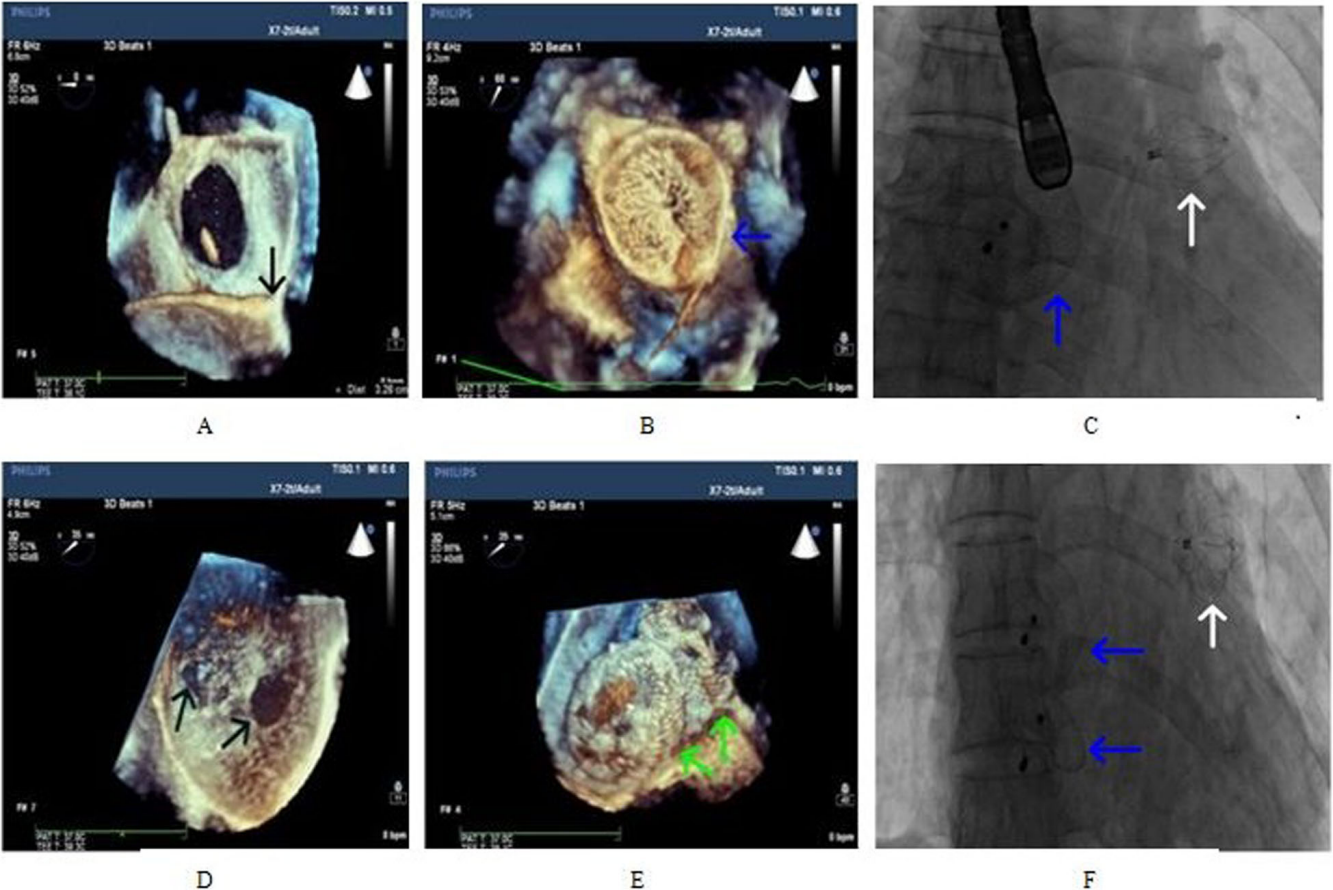
3D TEE imaging and X-ray imaging of successful LAAC and ASD occlusion for a large ASD patient and a double ASD patient with persistent AF (The black arrow indicates the LAA plug devices(**a**), dotted line indicates large ASD(**a**), blue arrow indicates the ASD plug devices(**b**). The blue arrow indicates the ASD plug devices, white arrow indicates the LAA plug devices(**c**); The black arrow indicates the double ASD(**d**), green arrow indicates the ASD plug devices(**e**); The blue arrow indicates the ASD plug devices, white arrow indicates theLAA plug devices(**f**))

Forty-nine patients successfully completed the one-stop occlusion. LAAC was performed first, followed by ASD/PFO occlusion; no operation-related complications occurred. The pressure in each cardiac cavity, LAA size, occluder size, and effect of occluder usage before one-stop occlusion in the two groups are shown in Table 2. A total of 26 ASD occluders were implanted in the 24 patients with ASD and AF (two double-hole ASD occluders were implanted in two patients), with an average diameter of 25.4 ± 8.5 mm. Conversely, 25 PFO occluders (including PF2525 in 22 patients and PF3030 in 3 patients) were implanted in the 25 patients with PFO and AF. In a patient with a large ASD and persistent AF, the diameter of ASD is 34 mm and the maximum width and depth of the LAA were 21 and 23 mm, respectively. The occlusion device was implanted successfully with the use of a 27-mm Watchman occluder and 44-mm ASD occlude (Fig. 3a, b and c). In one patient with double-hole ASD and persistent AF, the largest diameter of the LAA was 27 mm, and the largest depth was 31 mm; the occlusion was successful with the use of a 33-mm Watchman occluder. The diameter of the double-hole ASD was 11 and 13 mm, respectively; the spacing was 10.5 mm; and the occlusion was successful with the use of 22- and 24-mm ASD occluders (Fig. 3d, e and f).

**Table 2:** Comparison of LAA occlusion data

Patient characteristics	ASD group(*n* = 24)	PFO group(*n* = 25)	P
Cardiac Catheterization			
LAP (mmHg)	15.3 ± 3.1	14.5 ± 4.0	0.45
RAP (mmHg)	11.3 ± 2.8	10.6 ± 3.1	0.39
RVP (mmHg)	19.8 ± 4.2	16.3 ± 4.7	0.008
PAP (mmHg)	23.9 ± 4.4	21.2 ± 4.5	0.04
TEE			
LAA width (mm)	20.6 ± 2.9	19.8 ± 2.0	0.28
LAA depth (mm)	27.9 ± 2.7	27.1 ± 2.3	0.27
LAA angiography			
LAA width (mm)	19.7 ± 2.8	19.3 ± 1.8	0.53
LAA depth (mm)	27.2 ± 2.4	26.6 ± 2.3	0.37
LAA Occluder size (mm)	27.2 ± 3.6	26.8 ± 2.3	0.60
Compression ratio (%)	20.8 ± 5.1	19.9 ± 5.7	0.59
Residual shunt	2/24	2/25	0.966

### Follow-up visit

All patients were followed up for more than 1 year, with an average follow-up duration of 29.0 ± 12.1 months. Twenty-three patients took warfarin (average INR, 2.3 ± 0.2), and 26 patients took NOAC (dabigatran in 20 patients and rivaroxaban in 6 patients). One case of skin ecchymosis occurred after warfarin administration. The INR was 3.5. The ecchymosis disappeared after the warfarin dosage was adjusted. After 45–60 days, TEE showed that the position of the occluder was normal and that occluder thrombosis occurred in two patients (Fig. 1e) (warfarin was used in one and new anticoagulant in the other); the anticoagulant treatment scheme was then adjusted (the warfarin group switched to receiving a new anticoagulant and vice versa, and the INR was maintained at 2.0–3.0). TEE showed that the thrombus disappeared 6 months after operation (Fig. 1f). No stroke, TIA, or other thromboembolism occurred in all patients during the follow-up period.

### The influence of one-stop occlusion on cardiac morphology and structure

All patients completed the TEE examination at 3, 90 and 180 days after operation. Table 3 shows the comparison of the measurement results of the diameters of left atrium, right atrium, left ventricle, and right ventricle and LVEF between the two groups.

**Table 3:** Echocardiographic results of the diameters of left atrium, right atrium, left ventricle, and right ventricle and LVEF between the two groups

	ASD group(n = 24)				PFO group(n = 25)			
	Pre-operation	3 day	90 day	180 day	Pre-operation	3 day	90 day	180 day
LA (mm)	44.5 ± 7.2	44.1 ± 7.0(*p* = 0.13)	44.2 ± 5.8(*p* = 0.25)	44.3 ± 5.3(*p* = 0.39)	48.1 ± 7.83	47.9 ± 7.4(*p* = 0.26)	48.2 ± 7.3(*p* = 0.44)	48.4 ± 7.3(*p* = 0.34)
RA (mm)	49.5 ± 9.5	46.1 ± 7.4(*p* = 0.005)	44.1 ± 6.7(*p* = 0.0001)	43.1 ± 6.8(p<0.01)	44.0 ± 7.8	43.8 ± 7.5(*p* = 0.35)	44.1 ± 7.7(p = 0.41)	44.3 ± 7.7(*p* = 0.33)
LV (mm)	46.5 ± 5.0	46.7 ± 3.9(*p* = 0.30)	46.4 ± 4.5(*p* = 0.43)	46.6 ± 4.1(*p* = 0.41)	49.9 ± 6.5	49.8 ± 5.7(*p* = 0.32)	49.7 ± 6.5(p = 0.39)	50.2 ± 6.3(p = 0.34)
RV (mm)	27.3 ± 6.5	25.9 ± 5.4(*p* = 0.04)	24.3 ± 4.7(p<0.01)	23.2 ± 4.3(p<0.01)	21.9 ± 3.9	21.8 ± 3.9(*p* = 0.29)	21.8 ± 3.9(p = 0.41)	22.2 ± 3.7(p = 0.26)
LVEF(%)	58.6 ± 6.5	60.7 ± 3.9(*p* = 0.08)	62.6 ± 5.0(*p* = 0.01)	62.8 ± 5.3(*p* = 0.006)	55.3 ± 11.5	55.5 ± 10.2(*p* = 0.42)	56.7 ± 9.9(*p* = 0.11)	56.8 ± 9.9(*p* = 0.09)

In the ASD group, the diameters of the right atrium and right ventricle decreased at 3, 90 and 180 days after operation, and the LVEF significantly increased at 90 and 180 days after operation; in the PFO group, the diameters of the left atrium, left ventricle, right atrium, and right ventricle and LVEF had no significant difference.

## Discussion

ASD is a clinically common congenital heart disease, and AF is one of the most common cardiac arrhythmias. ASD shunts in the atrium allow blood flow from the left atrium to the right atrium, resulting in increased atrial size, elevated atrial wall pressure and stretching force, reconstruction of the anatomical structure of the atrium, and atrial electrical remodeling. Therefore, patients with ASD often experience AF complications. Moreover, the occurrence rate of AF in patients with ASD increases significantly with age [12]. Mandalenakis [17], a Swedish doctor, reviewed the follow-up data of 21,982 young patients with congenital heart disease and compared them with those of a normal population. During the 42 years of clinical follow-up, the risk of AF in the patients with congenital heart disease was 21.99 times higher than that in the normal population, among which active pulse narrowing, ASD, and PFO were all high-risk factors. Although the patients with congenital heart disease were divided into two groups independently, it was found that those with congenital heart disease were more likely to develop stroke when they had heart failure or AF, and the risk of stroke was six times higher than that in other patients with congenital heart disease.

Further, AF may still occur in some patients with ASD after surgical correction or transcatheter closure. Park [18] found through a follow-up study that transient atrial flutter or AF, short posterior inferior margin of ASD, and age greater than 48 years were predictors of new AF after ASD treatment. Nyboe [19] reported that among 1168 patients who underwent ASD occlusion, the risk of AF and stroke was higher than that of normal individuals, and there was no significant difference between repair and occlusion, at an average follow-up duration of 9.6 years. Therefore, in cases of ASD with AF, even if ASD is treated with radical operation (surgical repair or transcatheter closure), the persistent AF still becomes an important cause of thromboembolism complications, which require long-term oral anticoagulant therapy. With the continuous progress of RFCA techniques for the treatment of AF guided by CARTO system, there have been reports about combination of RFCA and ASD occlusion. However, this therapy is only available for patients that the duration of AF is short or the atrial enlargement is not obvious. Moreover, patients with AF who are treated with RFCA may relapse. Thus, it is not an ideal therapy.

PFO and AF are important causes of cardiogenic embolism. Although three randomized controlled studies published in 2012–2013 did not report an effective role in the prevention of recurrence of stroke of unknown cause [20–22], that is, PFO blocking is not better than drug treatment, further analysis shows that the prevention of recurrence of stroke is related to the use of blocking devices. Amplatzer PFO occluder is reportedly better than drug therapy in the prevention of stroke recurrence [23, 24]. In recent years, LAAC has been widely used in clinical practice as a new technique to prevent stroke caused by non-valvular AF. A number of randomized controlled clinical studies also show that the therapeutic effect is better than that of oral anticoagulants. In particular, the 5-year follow-up results of PREVAIL and PROTECT-AF showed that the risk of hemorrhagic stroke was lower than that with warfarin. At present, although there are few reports of ASD/PFO combined with AF, there is still a lack of systematic research and long-term follow-up results. This study of 49 adult patients with ASD/PFO and AF showed that one-stop occlusion was feasible. However, the treatment requires detailed attention to selection of indications, technical execution, postoperative management, and follow-up, which should be strictly adhered to.

More attention should be given to the following points when selecting the indications of one-stop occlusion: (1) the indications should be strictly grasped. Patients with ASD without interventional occlusion indications or cardiovascular abnormality warranting thoracotomy optimally should undergo operation and excision of the LAA. (2) It is very difficult to perform LAAC after ASD occlusion (it is difficult to puncture the atrial septum); thus, it is necessary to carefully evaluate whether it is necessary to perform LAAC before ASD occlusion. At present, a CHA_2_DS_2_VASc score of ≥2 points is required as a more recognized indication of LAAC. Our study also strictly followed this standard. However, we also excluded some patients with ASD and persistent AF during recruitment. The CHA_2_DS_2_VASc score was less than 2 points, while the left atrium was significantly enlarged; further, only the atrial defect was occluded, and anticoagulant treatment was then performed. At present, we are continuing to perform follow-up. If patients are willing to receive LAAC, we need to explore whether one-stop treatment can be performed simultaneously.

The technical execution of one-stop occlusion in patients with ASD and AF also had detailed specifications. It was necessary to conduct LAAC first, followed by ASD occlusion.

We should avoid the displacement of the LAA occluder in the course of ASD occlusion. For patients with large ASD, the pulmonary vein release method might be applied for the ASD occlusion, as the patient in the present study, in whom an ASD size of 34 mm was successfully plugged with a 44-mm plug device using the pulmonary vein release method. The LAA occluders were not impacted. However, for double ASD patients, it is necessary to place two occluders. When the application of the pulmonary vein release method is inconvenient, more caution must be practiced, as pressure on the LAA occluders might cause them to disconnect, which should be avoided. In our double ASD case, two occluders with diameters of 22 and 24 mm were placed. Under strict TEE monitoring, the operation was completed without any complication, indicating that with strict monitoring and careful operative technique, one-stop occlusion is safe. In addition, although atrial septum puncture for LAAC is not necessary for ASD patients with persistent AF, it appears to be simpler. However, due to the larger ASD, the sheathing canal of LAA occluders is easy to sway and challenging to fix. Thus, on the contrary, it increases the operative difficulty index. Operators should have sufficient experience to ensure the stability of the sheathing canal and avoid serious complications.

After LAAC, antithrombotic therapy and device-related thrombus (DRT) have become some of the clinical issues of concern. Fauchier [25] reviewed the data of 469 patients with AF who received LAAC in eights centers in France from 2012 to 2017. The incidence rate of Watchman occluder DRT was 4.8% (13/272), which is consistent with the rate of 4.1% (2/49) found in our study. They concluded that the occurrence of DRT after LAAC was closely related to the selection of postoperative antithrombotic therapy and different blocking devices. At present, there is no consensus on the antithrombotic therapy plan within 6 months after LAAC. The previous research of the research group confirmed that the three plans of incorporating new anticoagulant, warfarin, and dual antiplatelet therapy after LAAC are feasible [26]. Two or three occluders were implanted in patients with ASD/PFO combined with AF for one-stop occlusion. Based on the previous experience on structural heart disease occlusion and the scheme after LAAC, anticoagulation therapy is recommended. Therefore, we compared the differences between NOAC and warfarin in two postoperative anticoagulant regimens and concluded that it is feasible to use NOAC or warfarin after operation for patients with ASD/PFO combined with AF. In this study, we found that by changing to other anticoagulants and prolonging the anticoagulant time in two patients with DRT, the DRT was resolved; this further stressed that after one-stop occlusion, the prognosis is generally good, as long as the regular antithrombotic treatment is maintained, even if DRT occurs.

### Limitations

There are some limitations in our study that need to be considered. Firstly, because the technology of one-stop occlusion is difficult, accumulation of extensive experience is necessary. In addition, the costs of one-stop occlusion are covered by some types of medical insurance, and others are transferred to patients directly. So some patients may not be able to afford the costs. Finally, further studies are required to determine the long-term safety and efficacy of this approach.

## Conclusion

In summary, suitable indications should be selected, and strict operative specifications are necessary for the treatment of patients with ASD and AF using one-stop occlusion. Reinforced monitoring and follow-up visits after operation are also critical. Given these requirements, one-stop occlusion is safe and effective for the treatment of adult patients with ASD/PFO and AF. It is also feasible to administer warfarin or NOAC after operation.

## Data Availability

The datasets used and/or analyzed during the current study are available from the corresponding author on reasonable request.

## Abbreviations

ASD: Atrial septal defect
PFO: Patent foramen ovale
AF: Atrial fibrillation
LAA: Left atrial appendage
LAAC: Left atrial appendage closure
RFCA: Radiofrequency catheter ablation
TEE: Transesophageal echocardiography
TTE: Transthoracic echocardiography
DRT: Device-related thrombus
NOAC: Novel Oral anticoagulants
